# Nicotine and Cotinine Inhibit Catalase and Glutathione Reductase Activity Contributing to the Impaired Osteogenesis of SCP-1 Cells Exposed to Cigarette Smoke

**DOI:** 10.1155/2018/3172480

**Published:** 2018-11-06

**Authors:** Romina H. Aspera-Werz, Sabrina Ehnert, Daniel Heid, Sheng Zhu, Tao Chen, Bianca Braun, Vrinda Sreekumar, Christian Arnscheidt, Andreas K. Nussler

**Affiliations:** Department of Traumatology, University of Tübingen, Schnarrenbergstraße 95, 72076 Tübingen, Germany

## Abstract

Cigarette smoking has been identified as a major risk factor for osteoporosis decades ago. Several studies have shown a direct relationship between cigarette smoking, decreased bone mineral density, and impaired fracture healing. However, the mechanisms behind impaired fracture healing and cigarette smoking are yet to be elucidated. Migration and osteogenesis of mesenchymal stem/stromal cells (MSCs) into the fracture site play a vital role in the process of fracture healing. In human nicotine, the most pharmacologically active and major addictive component present in tobacco gets rapidly metabolized to the more stable cotinine. This study demonstrates that physiological concentrations of both nicotine and cotinine do not affect the osteogenic differentiation of MSCs. However, cigarette smoke exposure induces oxidative stress by increasing superoxide radicals and reducing intracellular glutathione in MSCs, negatively affecting osteogenic differentiation. Although, not actively producing reactive oxygen species (ROS) nicotine and cotinine inhibit catalase and glutathione reductase activity, contributing to an accumulation of ROS by cigarette smoke exposure. Coincubation with N-acetylcysteine or L-ascorbate improves impaired osteogenesis caused by cigarette smoke exposure by both activation of nuclear factor erythroid 2-related factor 2 (Nrf2) signaling and scavenging of ROS, which thus might represent therapeutic targets to support fracture healing in smokers.

## 1. Introduction

Smoking is the most common method of consuming tobacco which is the most popular substance smoked. For that, tobacco is combusted and the smoke with the active substances is inhaled. Smoking cigarettes represents a major health risk, which increases morbidity and mortality. It affects the whole human body and is linked to various health disorders. Deleterious effects of cigarette smoking on bone integrity have been shown, with a positive correlation between the number of cigarettes smoked per day and the years of exposure. Furthermore, smoking affects patients submitted to orthopedic surgery negatively by delaying the fracture healing, increasing the frequency of complications, and prolonging the hospital stay [1, 2].

Over 150 of the 6000 molecular species present in cigarette smoke have been identified as toxic compounds [3, 4]. It is still not completely elucidated which of these compounds are responsible for the negative effects of smoking on bone metabolism and fracture healing. Nicotine is the most pharmacologically active component present in tobacco which directly and indirectly affects cellular metabolism. Several studies have shown dose-dependent positive effects of nicotine on proliferation and differentiation of mesenchymal stem/stromal cells (MSCs) [5, 6]. However, the concentration of nicotine in these *in vitro* studies was much lower than the concentration found in blood samples from smokers [5]. Other studies revealed negative effects of nicotine on MSC proliferation as well as differentiation [7, 8]. Therefore, the effect of nicotine on the osteogenic differentiation of MSCs still remains unclear.

Cotinine is the most important metabolite of nicotine. 70–80% of nicotine is converted to cotinine in the liver. This metabolite is also present in the blood from smokers, with an average of 250–300 ng/ml cotinine which reaches higher blood levels than nicotine (50–100 ng/ml), which might be due to the longer half-life of cotinine (nicotine 2 h, cotinine 16 h) [9].

Recently, we reported that oxidative stress induced by cigarette smoke extract (CSE) [10] could be one of the responsible factors for the impaired osteogenic differentiation of SCP-1 cells. Coincubation with the antioxidant resveratrol protected the SCP-1 cells from the CSE deleterious effect [11]. However, the underlying mechanisms are not completely understood.

Nuclear factor erythroid-2-related factor-2 (Nrf2) signaling is known as a major mechanism in the cellular defense against oxidative stress which is activated in response to stress conditions [12]. In an unstressed condition, Nrf2 is sequestered in the cytoplasm by Kelch-like ECH associating protein 1 (Keap-1) [13] which favors its proteasomal degradation. Under stress conditions, Keap-1 changes its structure by stabilizing its thiol groups, which interferes with its binding to Nrf2. Free in the cytoplasm, Nrf2 is activated [14] and translocates into the nucleus, where it binds to the antioxidant response element (ARE) in the promoter region of genes, e.g., antioxidative enzymes and genes involved in glutathione (GSH) homeostasis, regulating their expression. Some studies in mice have shown that disruption of Nrf2 impairs the induction of cellular defense pathways and increases the negative effects of oxidative stress induced by cigarette smoke [15]. Moreover, upregulating Nrf2 signaling by knockdown of Keap-1 increases antioxidative defense and diminishes lung injury caused by smoking [16]. However, there are controversial findings on the roles of antioxidant signaling pathways on bone metabolism under oxidative stress. On the one hand, it was shown that MC3T3 cells exposed to H_2_O_2_ activation of Nrf2 signaling negatively affect osteogenic differentiation—a mechanism inhibited by N-acetylcysteine (NAC) [17]. On the other hand, deletion of Nrf2 in bone tissue leads to a poor bone mineral density not only due to increased osteoclast activity but also because of a lack of functional osteoblasts [18, 19].

Up to now, it is not known if and how nicotine and cotinine affect the osteogenic differentiation of MSCs. Therefore, the aim of the present study was to evaluate the effect of nicotine and cotinine on MSCs during osteogenic differentiation and, furthermore, to investigate which type of reactive oxygen species (ROS) is induced by CSE, nicotine, or cotinine and how these substances affect the cell response to oxidative stress.

## 2. Materials and Methods

Anti-acetylated-*α*-tubulin, anti-SOD-1, and anti-rabbit HRP-labeled secondary antibodies were obtained from Santa Cruz (SC-23950, SC-11407, and SC-2004; Heidelberg, Germany). Anti-GAPDH antibody was obtained from Sigma-Aldrich (G9545; Munich, Germany). Anti-phospho-p38 MAPKinase and anti-catalase antibodies were obtained from Cell Signaling (Frankfurt am Main, Germany). Anti-phospho-Nrf2 antibody was obtained from Abcam (ab76026; Cambridge, United Kingdom). Alexa Fluor 448-labeled secondary antibody was obtained from Invitrogen (Karlsruhe, Germany). N-Acetylcysteine (NAC) and nicotine were obtained from Carl Roth (Karlsruhe, Germany). L-Ascorbic acid was obtained from Sigma-Aldrich (Darmstadt, Germany). Cotinine was obtained from Alfa Aesar (Karlsruhe, Germany).

### 2.1. Generation of Cigarette Smoke Extract (CSE)

CSE was freshly prepared for every experiment. In total, the smoke of two commercial cigarettes (Marlboro, Philip Morris, New York City, USA) containing 0.8 mg nicotine and 10 mg tar each was continuously bubbled through a 50 ml prewarmed SCP-1 differentiation medium (0% FCS) in a standard gas wash bottle, as described before [11]. The CSE was normalized by its optical density at 320 nm (OD_320_), with an OD_320_ of 0.7 considered 100% CSE [20]. After sterile filtration (0.22 *μ*m pore filter), the CSE was diluted with SCP-1 differentiation medium to receive 5% *V*/*V* CSE, which corresponds to exposures associated with smoking up to 10 cigarettes/day [21].

### 2.2. Culture and Osteogenic Differentiation of SCP-1 Cells

Human immortalized mesenchymal stem cells (SCP-1 cells, provided by Dr. Matthias Schieker [22]) were cultured in MEM alpha medium (10% *V*/*V* FCS, 100 U/ml penicillin, and 100 mg/ml streptomycin) in a water-saturated atmosphere of 5% CO_2_ at 37°C [23]. SCP-1 cells were osteogenically differentiated for 21 days in MEM alpha medium (1% *V*/*V* FCS, 100 U/ml penicillin, 100 mg/ml streptomycin, 200 *μ*M L-ascorbate-2-phosphate, 10 mM *β*-glycerol-phosphate, 25 mM HEPES, 1.5 mM CaCl_2_, and 100 nM dexamethasone). The medium was changed every 3–4 days.

### 2.3. Resazurin Conversion Assay

Cell viability (mitochondrial activity) was measured by Resazurin conversion assay. Briefly, cells were covered with 0.0025% *W*/*V* Resazurin in PBS. After 30 min incubation at 37°C, the resulting Resorufin fluorescence was measured (excitation = 544 nm/emission = 590 nm) as described [24, 25]. The incubation time was optimized based on the high metabolic activity of SCP-1 cells.

### 2.4. Sulforhodamine B (SRB) Staining to Assess Total Protein Content

Total protein content was determined by SRB staining of ethanol-fixed (1 h at −20°C) cells. Cells were stained with 0.4% *W*/*V* SRB (in 1% *V*/*V* acetic acid) for 20 min at ambient temperature. Cells were washed 4–5 times with 1% acetic acid to remove unbound SRB. Bound SRB was resolved with 10 mM unbuffered TRIS solution (pH ~10.5). Resulting absorption (*λ* = 565 nm) was determined with a plate reader [26].

### 2.5. Alkaline Phosphatase (AP) Activity Assay

AP activity was measured as an early osteogenic marker. Briefly, cells were incubated with AP reaction buffer (0.2% *W*/*V* 4-nitrophenyl-phosphate, 50 mM glycine, 1 mM MgCl_2_, 100 mM TRIS, and pH 10.5) for 30 min at 37°C. Formed 4-nitrophenol was determined photometrically (*λ* = 405 nm) as described and normalized to relative cell numbers by SRB staining. Changes in AP activity are displayed relative to untreated cells [24, 25].

### 2.6. Alizarin Red Staining

Matrix mineralization was measured as a late osteogenic marker. Cells were fixed with ice-cold ethanol for 1 h. After washing with tap water, cells were incubated with 0.5% *W*/*V* Alizarin Red solution (pH 4.0) for 30 min at ambient temperature. After 3 additional washing steps, the resulting staining (red) was assessed microscopically. After resolving the stain with 10% *W*/*V* cetylpyridinium chloride, Alizarin Red staining was quantified photometrically at *λ* = 562 nm [24, 25].

### 2.7. Immunofluorescence Staining

Cells were fixed with 4% *V*/*V* paraformaldehyde solution and permeabilized with 0.2% *V*/*V* Triton-X-100 for 10 min each. Unspecific binding sites were blocked with 5% *W*/*V* BSA for 1 h. Incubation with primary antibodies (1 : 100) was performed overnight at 4°C, followed by incubation with ALEXA488 labeled secondary antibodies (1 : 2.000) for 1 h. Images were taken with a fluorescence microscope (EVOS FL AF 4301, Life Technologies, Darmstadt, Germany). The excitation and emission wavelengths were used as specified by the manufacturer. Pictures were analyzed using the ImageJ software (line tool) (National Institute of Health, Bethesda, USA) by 4 independent investigators in a blinded fashion. Based on the microscopic pictures taken, cilia length was determined by the maximum intensity projection method [27].

### 2.8. Determination of ROS Levels

To measure the formation of ROS, different fluorescent probes were used [28]:

- For the most unspecific 2′,7′-dichlorofluorescein-diacetate (DCFH-DA) assay, cells were incubated with 10 *μ*M DCFH-DA for 25 min at 37°C. After washing twice with PBS, cells were stimulated with CSE according to the experimental setup. As positive control, SCP-1 cells were stimulated with 0.01% *V*/*V* (882 *μ*M) H_2_O_2_. After 0, 5, 10, and 15 min, the increase in fluorescence (excitation = 485 nm/emission = 520 nm) was detected by a plate reader, representing levels of ^·^O_2_^−^, H_2_O_2_, HO^·^, and ONOO^−^ [29]. To trap the ROS, cells were coincubated with either 25 *μ*M *α*-tocopherol (^·^O_2_^−^_i_), 10 mM sodium-pyruvate (H_2_O_2i_), 250 mM DMSO (HO^·^_i_), or 100 *μ*M uric acid (ONOO^−^_i_) [28]
- To determine ^·^O_2_^−^ levels, cells were incubated with 10 *μ*M dihydroethidium (DHE) for 25 min at 37°C. After washing twice with PBS, cells were stimulated with CSE according to the experimental setup. As negative control (assay specificity), SCP-1 cells were stimulated with 0.01% *V*/*V* (882 *μ*M) H_2_O_2_. After 0, 5, 10, and 15 min, the increase in fluorescence (excitation = 544 nm/emission 590 nm) was detected by a plate reader. The slope of the linear part of the curve, resembling the product formation rate, was calculated. Cellular localization of the fluorescence was confirmed by fluorescence microscopy

### 2.9. Determination of Total Glutathione

The total GSH measurement was performed according to the Ellman assay: after stimulation, protein precipitation of cellular lysates was carried out with 3% *W*/*V* m-phosphoric acid. The protein samples were reneutralized with 5 mM EDTA in 0.1 M potassium phosphate buffer (pH = 7.4), and the total GSH was determined. For the determination, 20 *μ*l of sample was incubated for 30 seconds with 120 *μ*l of a mixture (1 : 1) of 1.68 mM 5,5′-dithiobis-(2-nitrobenzoic acid) and 2.5 U/ml glutathione reductase in 0.1 M potassium phosphate buffer. Then, 60 *μ*l of NADPH 0.8 mM was added and absorbance was measured at *λ* = 412 nm for 15 min [30].

### 2.10. Western Blot Analysis

Cells were lysed in freshly prepared ice-cold RIPA buffer. 30 *μ*g total protein was separated by SDS page and transferred to nitrocellulose membranes. Membranes were blocked with 5% *W*/*V* BSA for 1 h. After overnight incubation with primary antibodies (1 : 1.000) at 4°C, membranes were incubated with the corresponding peroxidase-labeled secondary antibodies (1 : 10.000) for 2 h at ambient temperature. For signal development, membranes were incubated for 1 min with ECL substrate solution. Chemiluminescent signals were quantified using the ImageJ software [24].

### 2.11. Catalase Activity Assay

The catalase activity was measured with the fluorometric catalase activity kit OxiSelect (Cell Biolabs, San Diego, CA, USA) according to the manufacturer's instructions. Fluorescence was measured at 544 nm (*λ*ex) and 590 nm (*λ*em) [31].

### 2.12. Superoxide Dismutase Activity Assay

In order to measure the SOD activity, SOD from HepG2 cells and a commercially available kit (Sigma-Aldrich, Taufkirchen, Germany) were used according to the manufacturer's protocol. Absorbance was measured at 450 nm every 5 min over 30 min [31].

### 2.13. Glutathione Peroxidase (GPx) Activity Assay

The measurement of GPx activity was performed using cumene hydroperoxide as a substrate for GPx. 5 *μ*l of 1 U/ml GPx was mixed with 5 *μ*l of each sample, 15 *μ*l of 4 mM NADPH, and 75 *μ*l of GPx assay solution (1.33 U/ml glutathione reductase, 1.33 mM L-glutathione reduced in 0.05 mM potassium phosphate buffer (pH = 7.0) containing 1.1 mM EDTA and 1.1 mM NaN_3_) and incubated at RT for 5 min. Then, 10 *μ*l of 15 mM cumene hydroperoxide solution was added and the decrease in absorbance at *λ* = 340 nm was measured within a 15 min time interval [30, 32].

### 2.14. Glutathione Reductase (GR) Activity Assay

The GR activity was measured by the increase in the absorbance due to the reduction of 5′-dithiobis-(2-nitrobenzoic acid) to 5-thio-(2-nitrobenzoic acid). 2.5 *μ*l of 1 U/ml GR and 2.5 *μ*l of each sample were mixed with 185 *μ*l reaction mixture (0.8 mM DTNB, 0.1 mM NADPH, and 1 M EDTA (1 M) in 0.2 M potassium phosphate buffer (pH = 7.5)) and 10 *μ*l of 20 mM L-glutathione (oxidized). Then, the increase in absorbance at *λ* = 412 nm was measured of a time interval of 15 min [30, 33].

### 2.15. Statistics

Results are expressed as the bar chart (mean ± SEM) of at least 4 independent experiments (*N* ≥ 4) measured as triplicate or more (*n* ≥ 3). Data sets were compared by the Kruskal-Wallis H test followed by Dunn's multiple comparison test (GraphPad Software Inc., La Jolla, CA, USA). *p* < 0.05 was taken as the minimum level of significance.

## 3. Results

### 3.1. Effect of Nicotine and Its Primary Metabolite Cotinine on Osteogenic Differentiation of SCP-1 Cells

**Figure 1:**
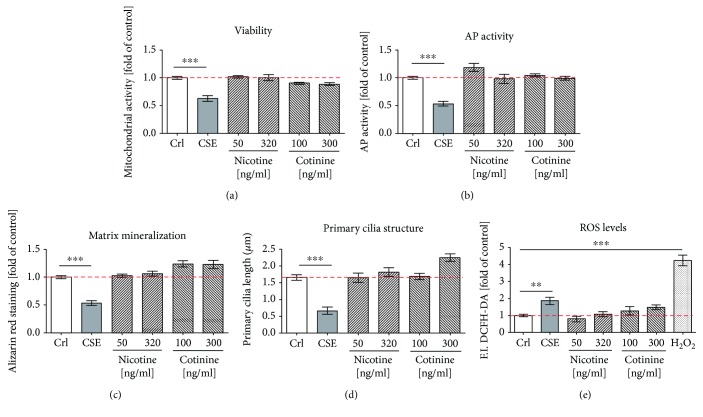
Nicotine and cotinine do not affect osteogenic differentiation of SCP-1. SCP-1 cells were osteogenically differentiated with nicotine (50 and 320 ng/ml) or its primary metabolite cotinine (100 and 300 ng/ml). Cell viability by Resazurin conversion (a) and AP activity (b) was measured on day 14. (c) Matrix mineralization was evaluated by Alizarin red after 21 days. (d) Primary cilium length was measured on day 21. (e) DCFH-DA assay was used to detect ROS in SCP-1 cells exposed to nicotine and cotinine. 0.01% *V*/*V* H_2_O_2_ was used as a positive control. Each experiment was conducted at least four times independently with triplicate. The statistical significance was determined by the Kruskal-Wallis H test followed by Dunn's posttest. Data are represented as the mean ± SEM, and the significance was represented as ^∗∗∗^ *p* < 0.001 vs the control group.

In order to determine which components present in cigarette smoke are responsible for impaired osteogenic differentiation of MSCs exposed to CSE [11], SCP-1 cells were treated with nicotine and its first metabolite cotinine. Nicotine and cotinine were applied to the cells at concentrations ranging from 50 ng/ml to 320 ng/ml and 100 ng/ml to 300 ng/ml, respectively. These concentrations were chosen based on reported blood levels of nicotine and cotinine from smokers and the calculated amount from our CSE. Nicotine concentration in arterial blood from smokers ranks between 20 and 60 ng/ml and rises up to 100 ng/ml after smoking one cigarette [9]. The average of cotinine in blood from smokers is 250–300 ng/ml [9]. The theoretical concentration of nicotine and cotinine in our CSE is 160 ng/ml and 150 ng/ml, respectively [11]. Therefore, SCP-1 cells were osteogenically differentiated for 21 days, in the presence of nicotine and cotinine, with concentrations up to 320 ng/ml and 300 ng/ml, respectively. Based on our previous work, 5% CSE was used as control. After osteogenic differentiation, effects on the mitochondrial activity (an indirect indicator of viability and proliferation) were measured by Resazurin conversion (Figure 1(a)). Mitochondrial activity of SCP-1 cells exposed to nicotine and cotinine was not significantly affected (Figure 1(a)). The differentiation status of the SCP-1 cells was evaluated by AP activity and matrix mineralization: AP activity, an early marker of osteogenic differentiation [34], after 14 days and the production of matrix mineralization, a late marker of osteogenic differentiation [34], after 21 days. Similar to the viability of the cells, the AP activity and the matrix production were not affected by nicotine and cotinine (Figures 1(b) and 1(c)).

Primary cilia, a microtubule-based organelle, have been shown to play an important role in the initiation of osteogenic differentiation of MSCs and also in the maintenance of function in the differentiated cells [35]. Therefore, the primary cilium structure was assessed by immunofluorescence staining of acetylated *α*-tubulin on SCP-1 cells differentiated in the presence of nicotine or cotinine for 21 days. Primary cilia on SCP-1 cells exposed to CSE showed a reduction of 62.5% in the length. However, nicotine and cotinine exposure did not affect the primary cilia structure of SCP-1 cells (Figure 1(d)). Representative immunofluorescence staining pictures of primary cilia are shown in Supplementary Figure 1.

Since the production of ROS is one of the best known negative consequences of cigarette smoking, we were interested in evaluating the effect of nicotine and cotinine in ROS production by SCP-1 cells. After exposure to CSE, the production of ROS by SCP-1 cells significantly increased; however, ROS levels did not increase after nicotine exposure. Surprisingly, its first metabolite showed a slight (not significant) increase in ROS production by SCP-1 cells, which seemed to be dose-dependent, concluding that nicotine and its first metabolite, cotinine, are not the direct effectors inducing ROS production in SCP-1 cells exposed to CSE. However, this data does not exclude that both substances might interfere with the cells' antioxidative defense mechanisms and thus indirectly favor the accumulation of ROS in the presence of CSE.

### 3.2. CSE Induced Oxidative Stress by Increasing ^·^O_2_^−^ and Reducing GST Activity in SCP-1 Cells

**Figure 2:**
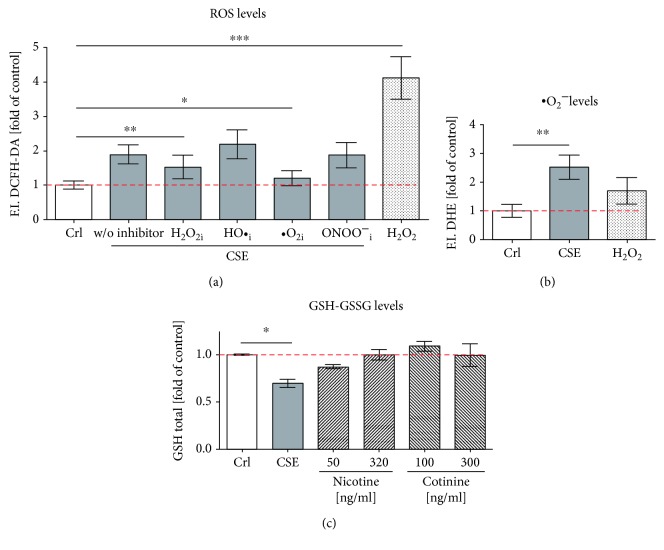
CSE induces oxidative stress with an increase in ^·^O_2_ and GSH reduction in SCP-1. SCP-1 cells were exposed to 5% CSE, and intracellular ROS and GSH levels were measured with different fluorescent probes: (a) DCFH-DA assay was used to detect ^·^O_2_^−^, H_2_O_2_, HO^·^, and ONOO^−^. To trap different ROS, SCP-1 cells were coincubated with 25 *μ*M *α*-tocopherol (^·^O_2_ i), 10 mM sodium-pyruvate (H_2_O_2_i), 250 mM DMSO (HO^·^i), or 100 *μ*M uric acid (ONOO^−^ i); (b) DHE assay was used to detect ^·^O_2_^−^, and (c) Ellman assay was used to detect total GSH levels. Results were normalized to control SCP-1 cells (Crl). 0.01% *V*/*V* H_2_O_2_ was used as positive control (a) or negative control (b) of the assays. Each experiment was conducted at least four times independently with triplicate. The statistical significance was determined by the Kruskal-Wallis H test followed by Dunn's posttest. Data are represented as the mean ± SEM, and the significance was represented as ^∗^ *p* < 0.05, ^∗∗^ *p* < 0.01, and ^∗∗∗^ *p* < 0.001 vs the control group.

In order to better identify the ROS formed by CSE exposure, a DCFH-DA assay with several radical scavengers was performed [28, 31]. Exposure to CSE significantly (2 fold) induced ROS levels, measured by DCFH-DA assay. Incubation with 25 *μ*M *α*-tocopherol, which traps ^·^O_2_^−^, significantly reduced ROS levels in SCP-1 cells exposed to CSE. However, scavengers of H_2_O_2_ (10 mM sodium-pyruvate), HO^·^ (250 mM DMSO), and ONOO^−^ (100 *μ*M uric acid) could not significantly reduce ROS levels in SCP-1 cells exposed to CSE (Figure 2(a)). In order to confirm that ^·^O_2_^−^ is induced by CSE in SCP-1 cells, a dihydroethidium (DHE) assay was performed. CSE exposure, but not nicotine and cotinine, significantly increased (2.5 fold) the level of ^·^O_2_^−^ (Figure 2(b)). Since GSH is the master antioxidant present in mammalian cells to prevent damage caused by ROS, the total GSH was measured by Ellman assay. SCP-1 cells exposed to CSE significantly decreased total GSH levels; nevertheless, nicotine and cotinine did not affect total GSH levels (Figure 2(c)). Thus, increased levels of ^·^O2^−^ and decreased total GSH affect the antioxidant capacity of SCP-1 cells exposed to CSE.

### 3.3. Antioxidants Rescued CSE-Impaired Osteogenesis in SCP-1 Cells

**Figure 3:**
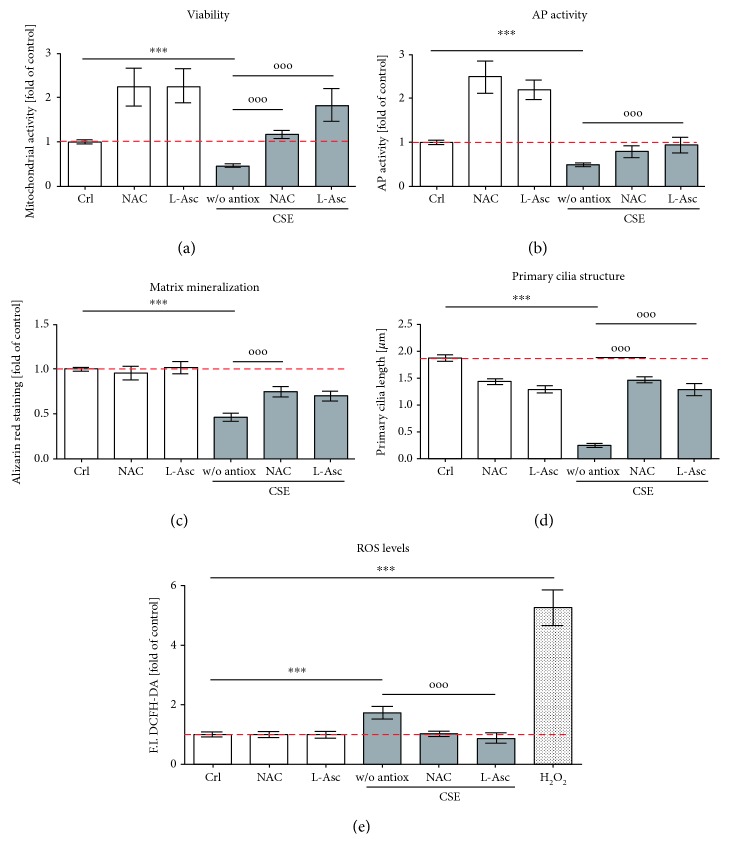
Antioxidants rescue CSE-impaired osteogenesis in SCP-1. SCP-1 cells were osteogenically differentiated with coincubation of antioxidants NAC 1 mM or L-Asc 200 *μ*M and CSE 5%. After 14 days of treatment, (a) the viability of the cells was measured by Resazurin conversion. The differentiation status of the cells was evaluated by (b) AP activity at day 14 and (c) Alizarin red staining at day 21. (d) Primary cilium length was measured at day 21. (e) DCFH-DA assay was used to detect ROS in SCP-1 cells exposed to 5% CSE and coincubation of antioxidants NAC 1 mM or L-Asc 200 *μ*M. 0.01% *V*/*V* H_2_O_2_ was used as positive control. Each experiment was conducted at least four times independently with triplicate. The statistical significance was determined by the Kruskal-Wallis H test followed by Dunn's posttest. Data are represented as the mean ± SEM, and the significance was represented as ^∗^ *p* < 0.05, ^∗∗^ *p* < 0.01, and ^∗∗∗^ *p* < 0.001 vs control and °*p* < 0.05, °°*p* < 0.01, and °°°*p* < 0.001 vs CSE.

The overproduction of ROS as well as the decrease of intracellular GSH, beyond the antioxidant scavenging capacity of the cells, causes oxidative stress that disrupts the primary cilia structure, which in turn impairs osteogenic differentiation of SCP-1 cells [11]. Previous studies have demonstrated the positive effects of NAC and L-ascorbate on osteogenesis [17, 36–42]. Therefore, to protect SCP-1 cells from oxidative stress generated by CSE, the effect of NAC and L-ascorbate during osteogenic differentiation with CSE was evaluated. In order to determine the concentrations of NAC and L-ascorbate that were not toxic to the cells, SCP-1 cells were osteogenically differentiated in the presence of NAC (1 mM–30 mM) or L-ascorbate (200 *μ*M, 1 mM) for 14 days. According to the mitochondrial activity and the total protein staining of the cells, the concentrations of 1 mM NAC and 200 *μ*M L-ascorbate were used in the following experiments (Supplementary Figure 2). NAC and L-ascorbate significantly increased the mitochondrial activity of SCP-1 cells after 14 days of coincubation with CSE (Figure 3(a)). Their addition upregulated the AP activity and the matrix mineralization of SCP-1 cells differentiated with CSE after 14 and 21 days, respectively (Figures 3(b) and 3(c)). NAC and L-ascorbate restored the primary cilium structure on SCP-1 cells that was altered by CSE exposure (Figure 3(d)). Moreover, treatment with NAC or L-ascorbate significantly decreased ROS levels in SCP-1 cells exposed to CSE (Figure 3(e)). These results suggest that antioxidant treatment enhances primary cilium integrity and improves the osteogenic differentiation of SCP-1 cells exposed to CSE by decreasing oxidative stress.

### 3.4. Nrf2 Signaling Was Activated by NAC and L-Ascorbate in SCP-1 Cells during Osteogenic Differentiation with CSE

**Figure 4:**
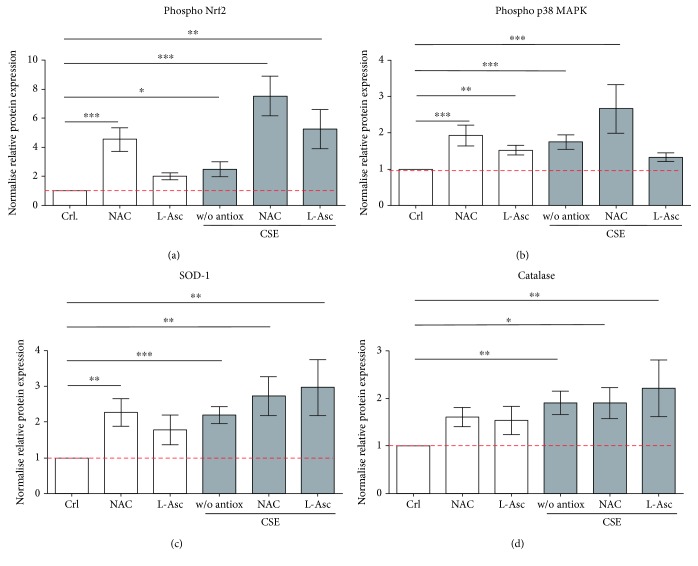
Nrf2-related signaling was activated by NAC and L-ascorbate in SCP-1 during osteogenic differentiation with CSE. SCP-1 cells were osteogenically differentiated with coincubation of antioxidants NAC 1 mM or L-Asc 200 *μ*M and CSE 5%. After 14 days of treatment, phosphorylated Nrf2 (a), p38 MAPKinase (b), SOD-1 (c), and catalase (d) protein expression levels were detected by Western blot. GAPDH was used as internal control. Each experiment was conducted at least three times independently with triplicate. The statistical significance was determined by the Kruskal-Wallis H test followed by Dunn's posttest. Data are represented as the mean ± SEM, and the significance was represented as ^∗^ *p* < 0.05, ^∗∗^ *p* < 0.01, and ^∗∗∗^ *p* < 0.001 vs the control group.

**Figure 5:**
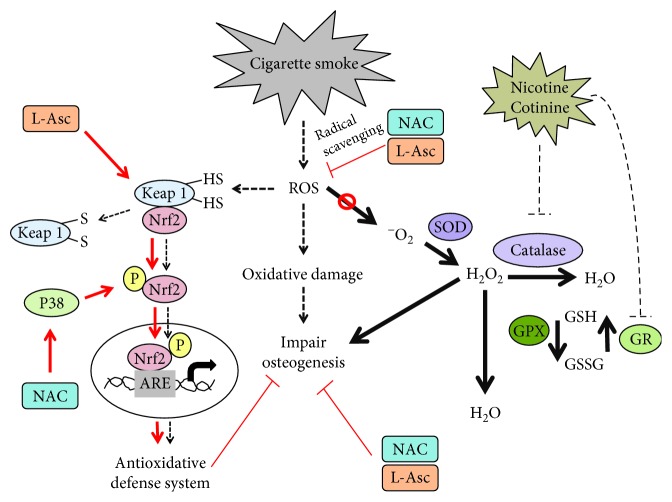
NAC and L-ascorbate enhance the osteogenic differentiation in SCP-1 cells exposed to CSE by activation of Nrf-2 signaling and through radical scavenging. Proposed mechanisms for oxidative stress impair osteogenic differentiation under CSE exposure and potential roles of antioxidant. High-level oxidative stress generated by CSE resulted in oxidative damage and impaired SCP-1 cells' osteogenic differentiation. ROS induced through CSE exposure can oxidize the Cys residues on Keap-1, leading to the conformational change and releasing Nrf2. Phospho-Nrf2 can translocate to the nucleus and activates the antioxidant response element (ARE) leading to an activation of antioxidant genes. However, activation of Nrf2 in CSE exposure cells may not be enough to protect the cells from the oxidative stress generated by CSE. NAC activates upstream p38 MAPKinase, which is required to activate Nrf2 and transactivate antioxidant genes that may reduce oxidative stress induced by CSE. L-Asc might act with thiol residues of Keap-1, increasing the levels of Nrf2 available. CSE inhibited catalase activity being not able to process H_2_O_2_. GR activity is also affected by CSE to a decrease of total GSH. NAC and L-Asc treatment decreased CSE-induced ROS production by increasing the biosynthesis of GSH via Nrf2 signaling and also by radical scavenging. CSE decreased the enzymatic activity of SOD and catalase, leading to accumulation of ^·^O_2_^−^ and H_2_O_2_ in the cells. Additionally, CSE decreased total GSH and decreased GR activity causing that there is no GSH availed. Therefore, GPx cannot catalyze the reduction of H_2_O_2_ to H_2_O. Nicotine and cotinine, despite not affecting the osteogenic differentiation of the cells, evidenced negative inhibitory effects on the enzymatic activity of catalase and GR. Nicotine and cotinine imbalance the antioxidative system contributing in part to the negative effects in the osteogenic differentiation of SCP-1 cell exposure to CSE.

Several studies have shown that redox-sensitive transcription factor Nrf2 plays an important role in cellular defense against oxidative stress by inducing the transcription of antioxidative enzymes [43, 44]. Therefore, the protective effect of the Nrf2 signaling pathway on SCP-1 cells, which were osteogenically differentiated and treated with antioxidants, was investigated. Western blot for phospho-Nrf2 and phospho-p38 MAPKinase was performed from SCP-1 cells differentiated with 5% CSE and 1 mM NAC or 200 *μ*M L-ascorbate. CSE exposure significantly increased the active form of Nrf2 in response to the oxidative stress (Figure 4(a)). Costimulation with NAC or L-ascorbate increased the levels of phospho-Nrf2 compared with SCP-1 cells exposed to CSE alone (Figure 4(a)), suggesting that the NAC and L-ascorbate protective effect is through an Nrf2-dependent mechanism and not only due to their radical scavenging properties. Besides, NAC coincubation with CSE significantly increased the levels of phospho-p38 MAPKinase (Figure 4(b)). Therefore, NAC-dependent activation of Nrf2 could be via the activation of protein kinases such as p38, causing phosphorylation and subsequent release of Nrf2 from its inhibitory protein Keap-1 (Figure 5). However, coincubation of CSE with L-ascorbate had no effect on phospho-p38 MAPKinase levels (Figure 4(b)), suggesting that L-ascorbate might react with the thiol residues in Keap-1, increasing the cellular availability of Nrf2 (Figure 5). Since the activation of the transcription factor Nrf2 was induced by antioxidant treatment, it was interesting to investigate the expression of SOD-1 and catalase, the target enzymes of this pathway. SOD-1 catalyzes the dismutation of two molecules of ^·^O_2_^−^ to H_2_O_2_ and molecular oxygen O_2_ for further processing. Catalase catalyzes the reduction of H_2_O_2_ to water and O_2_, completing the detoxification process initiated by SOD-1 [45]. Similarly, to Nrf2 activation, SOD-1 and catalase protein expression was upregulated by NAC and L-ascorbate coincubation with CSE (Figure 4(c) and 4(d)). Thus, an increase of SOD-1 and catalase initiated by Nrf2 might be involved in the protective effect of NAC and L-ascorbate during osteogenic differentiation of SCP-1 cells exposed to CSE. Representative Western blot pictures are shown in Supplementary Figure 3.

### 3.5. CSE, Nicotine, and Cotinine Generated an Imbalance in the Antioxidative System

**Figure 6:**
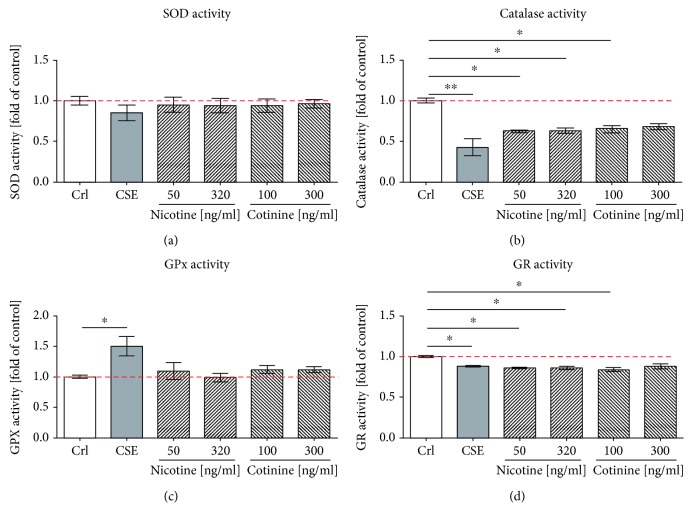
CSE generated an imbalance in the antioxidative system. Enzyme activities SOD (a), catalase (b), GPx (c), and GR (d) were determined with and without exposure to 5% CSE, 50 ng/ml or 320 ng/ml nicotine, and 100 ng/ml or 300 ng/ml cotinine. The enzymatic activity was expressed as the fold of control. Each experiment was conducted at least three times independently with triplicate. The statistical significance was determined by the Kruskal-Wallis H test followed by Dunn's posttest. Data are represented as the mean ± SEM, and the significance was represented as ^∗^ *p* < 0.05, ^∗∗^ *p* < 0.01, and ^∗∗∗^ *p* < 0.001 vs the control group.

Since CSE exposure activated Nrf2 signaling and induced the protein expression of antioxidative enzymes, it was interesting to evaluate the effect of CSE on the activity of enzymes involved in ^·^O_2_^−^ detoxification and GSH recycling. Thus, we investigated the effect of CSE, nicotine, and cotinine on the activity of the isolated enzymes involved in mitochondrial antioxidative defense, namely, SOD, catalase, GPx, and GR. Interestingly, the presence of CSE significantly decreased the activity of catalase (Figure 6(a)) and slightly decreased the enzymatic activity of SOD (Figure 6(b)). This might explain the observed accumulation of ^·^O_2_^−^ and H_2_O_2_ in the CSE-treated cells. However, GPx can also catalyze the reduction of H_2_O_2_ to H_2_O via oxidation of reduced GSH into its disulfide form. The presence of CSE significantly increased GPx enzymatic activity (Figure 6(c)). At the same time, the presence of CSE significantly decreased the total GSH (Figure 2(c)) and significantly decreased the GR activity (Figure 6(d)), causing that there is no GSH availed in cells exposed to CSE. Nicotine and cotinine, despite not affecting the osteogenic differentiation of the cells, evidenced significant inhibitory effects on the enzymatic activity of catalase and GR. Therefore, an imbalance in the antioxidative system induced by the most addictive substance, its first metabolite, and other molecules present in cigarette smoke affects the osteogenic differentiation of SCP-1 cells.

## 4. Discussion

Cigarette smoke contains more than 150 of 6000 molecular substances that are considered toxic compounds [3, 4]. Nicotine is known as the major active and addictive compound associated with smoking. The role of nicotine on osteogenic differentiation is still unclear, as different effects have been shown dependent on the concentration and exposure time. On the one hand, it has been shown that nicotine induces apoptosis in human osteoblasts via increased ROS levels [8]. Moreover, reduced matrix formation was observed with SaOS-2 cells exposed to nicotine for 14 days [7]. On the other hand, it has been reported that nicotine increases osteoblast activity in bone marrow stromal cells [5]. In our experiments, blood nicotine concentrations did not affect the osteogenic differentiation of SCP-1 cells; besides, its first metabolite cotinine also did not influence the osteogenic differentiation of SCP-1 cells. These results point out the negative role of ROS induced by CSE during osteogenic differentiation. In the clinic, smokers often show delayed fracture healing, increased frequency of complications, and prolonged hospital stays [1, 2]. This led to the assumption that devices that administer nicotine, e.g., e-cigarettes, tobacco heat systems, nicotine sprays, or tapes, could be a less harmful alternative for smoking in orthopedic patients.

In line with this assumption, our results clearly show that only CSE exposure, but not nicotine and cotinine, increases the levels of ^·^O_2_^−^ while reducing the total GSH available in the cells. From these results, we endorse previous suggestions that an imbalance in the antioxidative system could be responsible for the impaired osteogenesis observed in SCP-1 cells after CSE exposure [46–48].

Since we observed a significant reduction in total GSH levels and a significant increase in ROS levels upon CSE exposure, we decided to use NAC as a precursor for GSH to support the osteogenic differentiation of SCP-1 cells exposed to CSE. NAC is a derivative of the amino acid L-cysteine, in which the thiol (sulfhydryl) group exhibits antioxidative effects by scavenging free radicals [49]. Previous studies have shown the beneficial effects of NAC on osteogenic differentiation [37–39]. Moreover, NAC has been shown to reverse the negative effects of H_2_O_2_ on osteogenic differentiation of MC3T3-E1 cells [17]. We used L-ascorbate as a potential treatment strategy due to the fact that smokers present lower levels of L-ascorbate in the blood than nonsmokers [50, 51]. Therefore, smokers require a higher daily intake of L-ascorbate to reach similar blood levels than nonsmokers due to an increased metabolic demand and a defective L-ascorbate recycling [51, 52]. Furthermore, L-ascorbate is known for stimulating proliferation of osteoblasts as well as for possessing free radical scavenging properties [41, 42]. Besides its antioxidative properties, L-ascorbate has been shown to support bone formation by stimulating the production of collagen [40]. Indeed, concentrations of 1 mM NAC and 200 *μ*M L-ascorbate improved the differentiation of SCP-1 cells coincubated with CSE, resulting in an increased AP activity on day 14 of differentiation and an increased amount of mineralized matrix formed after 21 days of differentiation. The concentrations of L-ascorbate and NAC used in this study were higher than those found in blood plasma that can be obtained by oral supplementation (L-ascorbate 3 g every 4 h administered orally produces a peak plasma concentration of 200 *μ*M [53]; NAC 600 mg oral administration produces a peak plasma concentration of 15 *μ*M [54]). However, intravenous doses of L-ascorbate and NAC can produce plasma concentrations up to 800 *μ*M [53] and 121 *μ*M [55], respectively, suggesting the possibility of implementing intravenous applications of antioxidant treatment in smokers during the surgical reposition of fractures or even for a limited time during the process of fracture healing.

However, ROS levels are necessary to induce cellular responses. ROS induced by CSE exposure can oxidize the Cys residues on Keap-1, causing its conformational change and releasing Nrf2 for activation. The active phospho-Nrf2 can then translocate to the nucleus and, upon binding to the antioxidant response element (ARE), lead to the transcription of antioxidant enzymes like SOD, catalase, GPx, and others [56]. The impaired osteogenic function observed in the CSE-exposed SCP-1 cells suggests that a mechanism in this process fails or that the activation of Nrf2 in CSE-exposed cells may not be enough to protect the cells from the oxidative stress generated by CSE.

Addition of antioxidants like NAC and L-ascorbate partly restored the osteogenic differentiation of CSE-exposed SCP-1 cells. NAC and L-ascorbate treatment decreased CSE-induced ROS production. However, despite a strong activation of p38 MAPKinase signaling, which is required for the activation of Nrf2 [57, 58], NAC treatment could not significantly increase protein levels of activated Nrf2 or antioxidative enzymes, when compared to CSE exposure alone. Similarly, addition of L-ascorbate which might react with the thiol residues in Keap-1, increasing the Nrf2 availability, could not further increase the cellular levels of phospho-Nrf2 and antioxidative enzymes, when compared to CSE exposure alone.

In response to the oxidative stress induced by CSE exposure, we have shown that CSE can induce the antioxidant signaling pathways via its transcription factor phospho-Nrf2 and increase the protein expression of SOD-1 and catalase, suggesting that the cells react adequately to the stress stimuli. SOD and catalase are strongly upregulated during osteogenic differentiation of MSCs [59], suggesting key regulatory roles of these enzymes. In low amounts, H_2_O_2_ is involved in many cellular processes such as activation of signaling pathways involved in cell migration, proliferation, and differentiation [60]. Additionally, H_2_O_2_ has been shown to induce osteogenic differentiation of vascular smooth muscle cells through the increase of Runt-related transcription factor 2 (Runx2) a key transcription factor for osteogenesis [61]. However, with increasing amounts, H_2_O_2_ exerts damage to cellular macromolecules including proteins and DNA consequently, causing cell death [62–64].

Even so, SOD and catalase protein expression was induced upon stimulation with CSE, an impaired osteogenic function was still observed, suggesting that the function of the antioxidative enzymes during the differentiation with CSE fails. We could show that CSE as well as nicotine and cotinine strongly inhibits catalase activity, suggesting that the inhibitory effect of CSE on the enzymatic activity of catalase and SOD, contributed in part from nicotine and cotinine. This supposedly generated a feedback where the cells produce more antioxidative enzymes. However, in the presence of nicotine and cotinine, these enzymes cannot perform their function properly; thus, both substances seem to indirectly participate in the observed ROS accumulation by CSE. SOD being less affected converts ^·^O_2_^−^ and H_2_O_2_ which then accumulates in the cells as catalase is not able to further process the H_2_O_2_. Nicotine and cotinine, despite having no effect on osteogenic differentiation, also decreased catalase activity contributing to the negative effect observed with CSE, supporting previous results that demonstrated lower enzymatic activity of antioxidant enzymes in blood of smokers [65]. As GPx activity is not negatively affected by CSE, nicotine, or cotinine, it is conceivable that upregulation of GPx could compensate for the inhibition of catalase in the CSE-exposed cells. However, similar to catalase, GR activity is also affected by CSE, nicotine, and cotinine. Thus, these substances interfere with glutathione recycling, limiting the substrate for GPx and thus leading to a decrease in total GSH.

The observation that nicotine and cotinine inhibit catalase and GR function critically challenges the assumption that devices administering nicotine could be a less harmful alternative for smoking in orthopedic patients, as the trauma itself and the associated surgical intervention represent (oxidative) stress the body has to cope with.

## 5. Conclusions

In summary, our study shows for the first time that nicotine and cotinine do not directly affect osteogenic differentiation of MSCs; however, these compounds negatively affect the function of the antioxidative enzymes. Therefore, the most addictive compound present in cigarette smoke and its first metabolite contribute in part to the negative effects on osteogenic differentiation observed in MSC following CSE exposure.

## Data Availability

The data used to support the findings of this study are available from the corresponding author upon request.

## Supplementary Materials

Supplementary File 1 Supplementary Figure 1: nicotine and cotinine not affected hMSC primary cilia structure. Immunostained images of primary cilia in SCP-1 cells osteogenically differentiated at day 14 without CSE (a), with 5% CSE (b), 320 ng/ml nicotine (c), and 300 ng/ml cotinine (d) exposed are shown. Primary cilium is shown by acetylated *α*-tubulin (green) and nucleus by Hoechst staining.

Supplementary File 2 Supplementary Figure 2: toxicity test of NAC and L-Asc in hMSC. SCP-1 cells were osteogenically differentiated with increasing concentrations of NAC (a, b) and L-Asc (c, d). The toxicity was measure by Resazurin conversion (mitochondrial activity) (a–c) and SRB staining (total protein measure) (b–d) after 14 days. Each experiment was conducted at least four times independently with triplicate. The statistical significance was determined by the Kruskal-Wallis H test followed by Dunn's posttest. Data are represented as the mean ± SEM, and the significance is represented as ^∗∗∗^ *p* < 0.001 vs the control group.

Supplementary File 3 Supplementary Figure 3: protein expression analysis of antioxidative enzymes. Representative Western blot pictures from phosphorylated Nrf2, phosphorylated p38 MAPKinase, SOD-1, catalase, and GAPDH are shown. SCP-1 cells were osteogenically differentiated with coincubation of antioxidants NAC 1 mM or L-Asc 200 *μ*M and CSE 5%. After 14 days of treatment, protein expression level was detected.
