# Supplementary figures and images for: Nicotine and Cotinine Inhibit Catalase and Glutathione Reductase Activity Contributing to the Impaired Osteogenesis of SCP-1 Cells Exposed to Cigarette Smoke
Source: Oxid Med Cell Longev. 2018 Nov 6;2018:3172480. doi: 10.1155/2018/3172480 (PMC6250005; doi:10.1155/2018/3172480)

## Slide 1
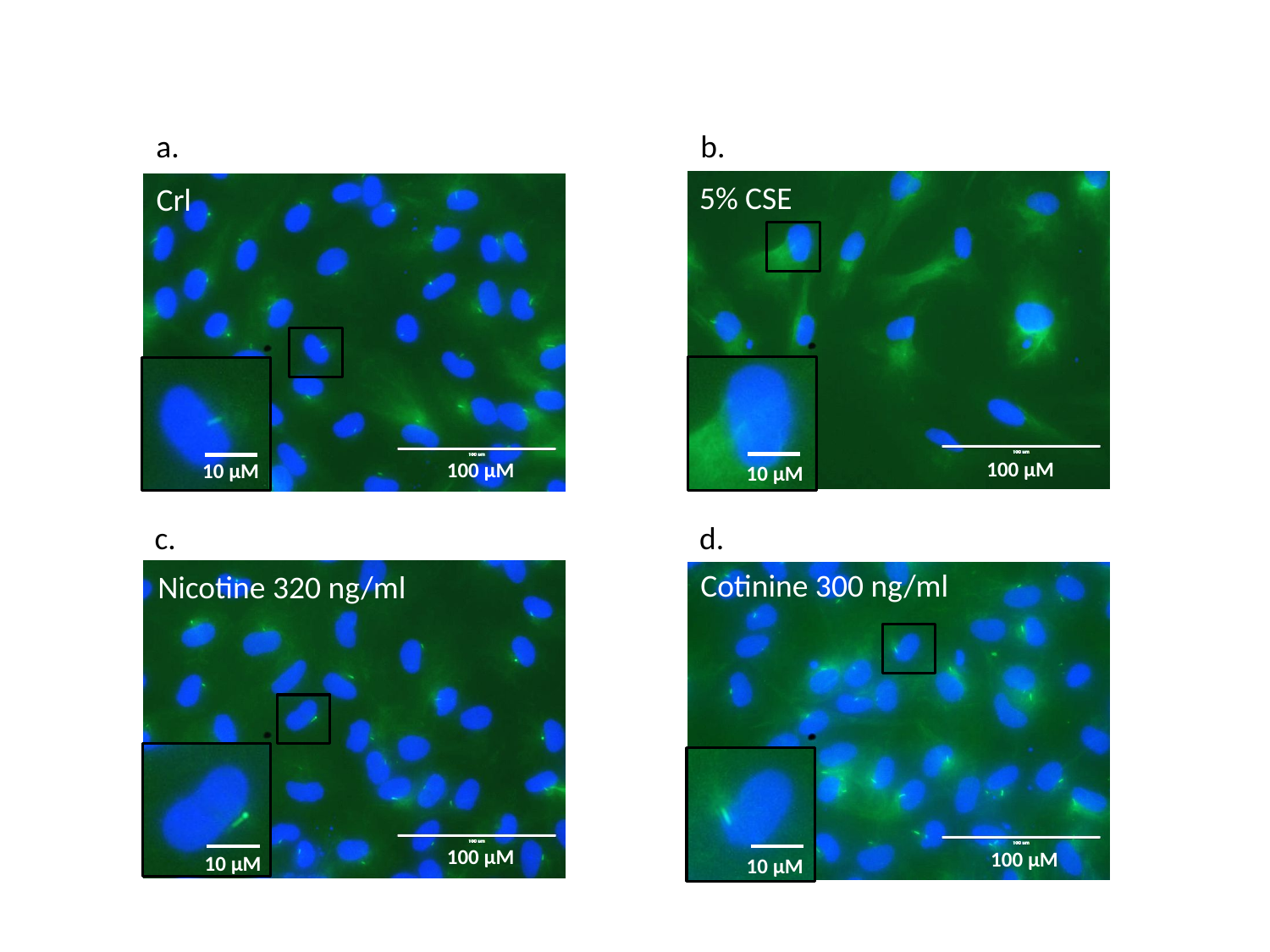

a.
Crl
10 µM
b.
5% CSE
10 µM
100 µM
100 µM
c.
Nicotine 320 ng/ml
10 µM
d.
Cotinine 300 ng/ml
10 µM
100 µM
100 µM

Supplement: Supplementary File 1 — Supplementary Figure 1: nicotine and cotinine not affected hMSC primary cilia structure. Immunostained images of primary cilia in SCP-1 cells osteogenically differentiated at day 14 without CSE (a), with 5% CSE (b), 320 ng/ml nicotine (c), and 300 ng/ml cotinine (d) exposed are shown. Primary cilium is shown by acetylated α-tubulin (green) and nucleus by Hoechst staining. [file 3172480.f1.pptx]

## Slide 1
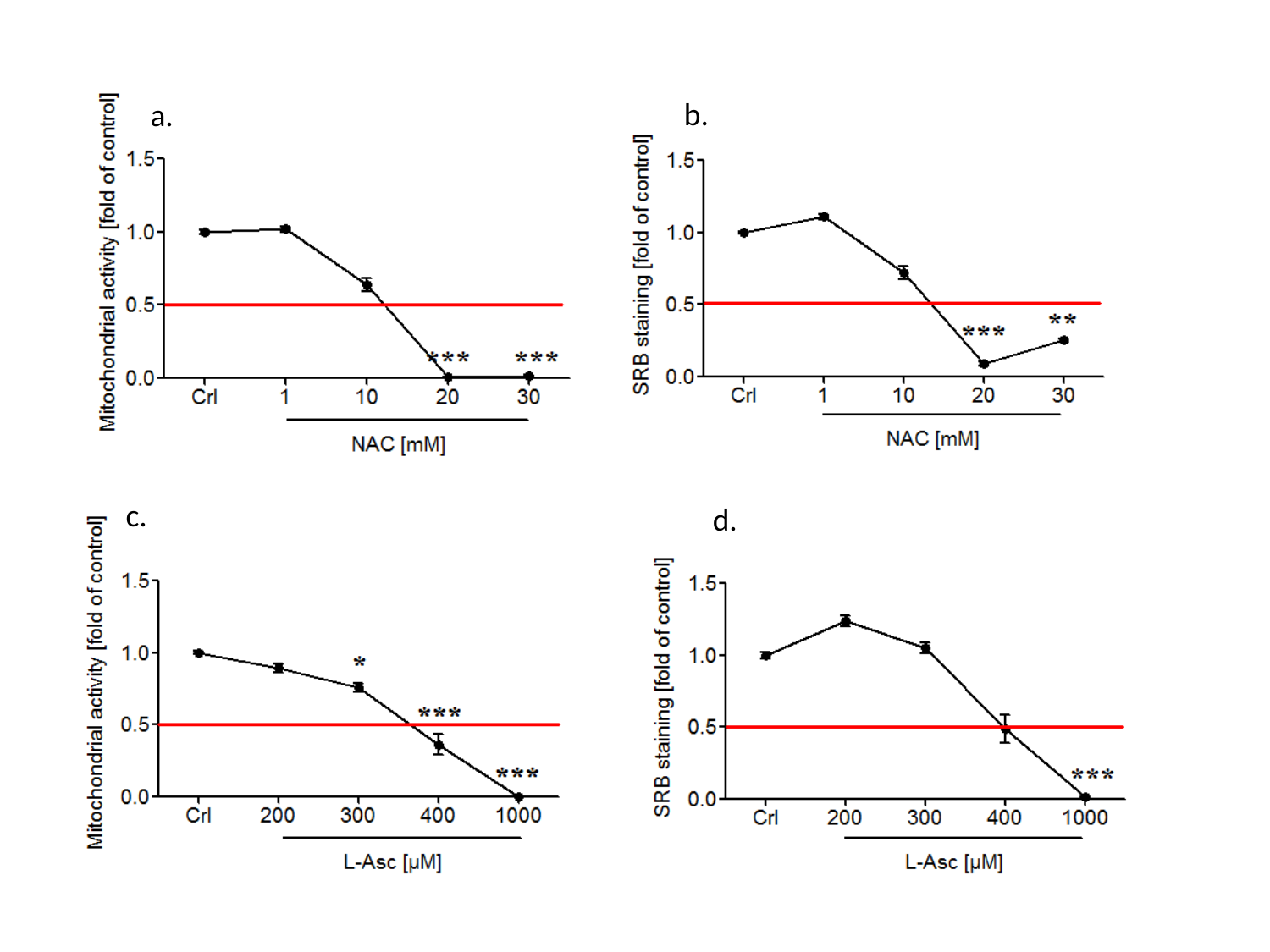

b.
a.
c.
d.

Supplement: Supplementary File 2 — Supplementary Figure 2: toxicity test of NAC and L-Asc in hMSC. SCP-1 cells were osteogenically differentiated with increasing concentrations of NAC (a, b) and L-Asc (c, d). The toxicity was measure by Resazurin conversion (mitochondrial activity) (a–c) and SRB staining (total protein measure) (b–d) after 14 days. Each experiment was conducted at least four times independently with triplicate. The statistical significance was determined by the Kruskal-Wallis H test followed by Dunn's posttest. Data are represented as the mean ± SEM, and the significance is represented as ∗∗∗ p < 0.001 vs the control group. [file 3172480.f2.pptx]
