# Supplementary material for: Nicotine and Cotinine Inhibit Catalase and Glutathione Reductase Activity Contributing to the Impaired Osteogenesis of SCP-1 Cells Exposed to Cigarette Smoke
Source: Oxid Med Cell Longev. 2018 Nov 6;2018:3172480. doi: 10.1155/2018/3172480 (PMC6250005; doi:10.1155/2018/3172480)
Supplement: Supplementary File 3 — Supplementary Figure 3: protein expression analysis of antioxidative enzymes. Representative Western blot pictures from phosphorylated Nrf2, phosphorylated p38 MAPKinase, SOD-1, catalase, and GAPDH are shown. SCP-1 cells were osteogenically differentiated with coincubation of antioxidants NAC 1 mM or L-Asc 200 μM and CSE 5%. After 14 days of treatment, protein expression level was detected. [file 3172480.f3.pptx]

## Slide 1
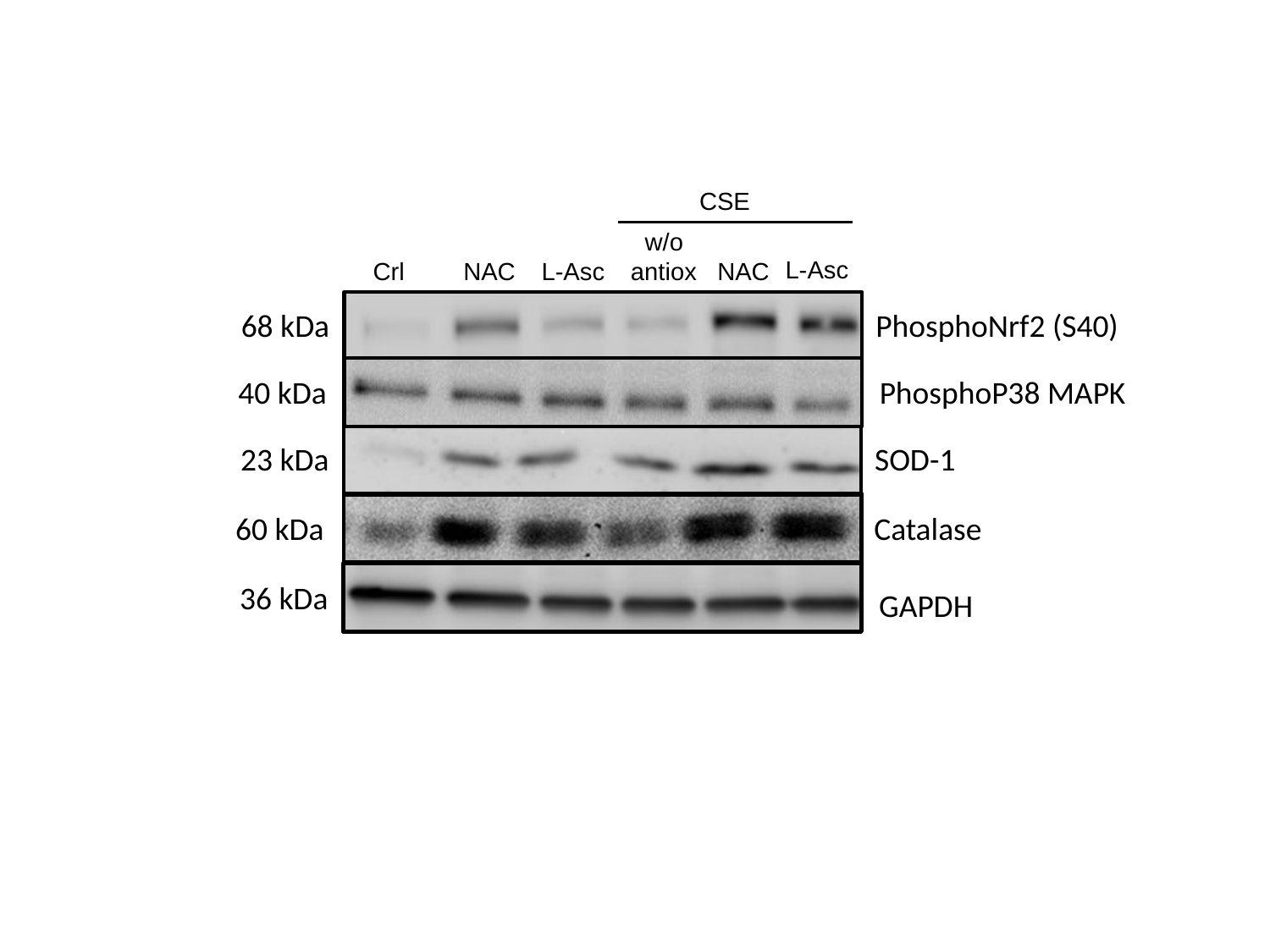

CSE
w/o
antiox
L-Asc
Crl
NAC
NAC
L-Asc
68 kDa
PhosphoNrf2 (S40)
PhosphoP38 MAPK
40 kDa
23 kDa
SOD-1
60 kDa
Catalase
36 kDa
GAPDH
